# Genetic Dissection and Identification of Candidate Genes for Salinity Tolerance Using Axiom^®^*CicerSNP* Array in Chickpea

**DOI:** 10.3390/ijms21145058

**Published:** 2020-07-17

**Authors:** Khela Ram Soren, Praveen Madugula, Neeraj Kumar, Rutwik Barmukh, Meenu Singh Sengar, Chellapilla Bharadwaj, Parbodh Chander Sharma, Sarvjeet Singh, Aditi Bhandari, Jogendra Singh, Satish Kumar Sanwal, Madan Pal, Sneha Priya P.R., Anita Mann, Someswar Rao Sagurthi, Shanmugavadivel PS, Kadambot H.M. Siddique, Narendra Pratap Singh, Manish Roorkiwal, Rajeev K Varshney

**Affiliations:** 1 ICAR-Indian Institute of Pulses Research (ICAR-IIPR), Kanpur UP 208024, India; sorenars@gmail.com (K.R.S.); meenu.sengar786@gmail.com (M.S.S.); psshanmugavadivel@gmail.com (S.P.); npsingh.iipr@gmail.com (N.P.S.); 2 Center of Excellence in Genomics & Systems Biology, International Crops Research Institute for the Semi-Arid Tropics (ICRISAT), Hyderabad 502324, India; pravi313@gmail.com (P.M.); r.barmukh@cgiar.org (R.B.); b.aditi@cgiar.org (A.B.); 3 Division of Genetics, ICAR-Indian Agricultural Research Institute (ICAR-IARI), Delhi 110012, India; neeraj0490@gmail.com (N.K.); drchbharadwaj@gmail.com (C.B.); madan_physio@iari.res.in (M.P.); snehapriya_reddy@yahoo.co.in (S.P.P.R.); 4 Department of Genetics, Osmania University, Hyderabad 500007, India; drsomeswar@osmania.ac.in; 5 ICAR-Central Soil Salinity Research Institute (ICAR-CSSRI), Karnal 132001, India; pcsharma.knl@gmail.com (P.C.S.); jogendra82@gmail.com (J.S.); satishsanwal@rediffmail.com (S.K.S.); Anita.mann@icar.gov.in (A.M.); 6 Department of Plant Breeding & Genetics, Punjab Agricultural University, Ludhiana 141004, India; sarvjeet62@pau.edu; 7 The UWA Institute of Agriculture, The University of Western Australia, Perth WA 6009, Australia; kadambot.siddique@uwa.edu.au

**Keywords:** chickpea, salinity, quantitative trait loci, stress susceptibility index (SSI), stress tolerance index (STI), candidate genes

## Abstract

Globally, chickpea production is severely affected by salinity stress. Understanding the genetic basis for salinity tolerance is important to develop salinity tolerant chickpeas. A recombinant inbred line (RIL) population developed using parental lines ICCV 10 (salt-tolerant) and DCP 92-3 (salt-sensitive) was screened under field conditions to collect information on agronomy, yield components, and stress tolerance indices. Genotyping data generated using Axiom^®^*CicerSNP* array was used to construct a linkage map comprising 1856 SNP markers spanning a distance of 1106.3 cM across eight chickpea chromosomes. Extensive analysis of the phenotyping and genotyping data identified 28 quantitative trait loci (QTLs) explaining up to 28.40% of the phenotypic variance in the population. We identified QTL clusters on CaLG03 and CaLG06, each harboring major QTLs for yield and yield component traits under salinity stress. The main-effect QTLs identified in these two clusters were associated with key genes such as calcium-dependent protein kinases, histidine kinases, cation proton antiporter, and WRKY and MYB transcription factors, which are known to impart salinity stress tolerance in crop plants. Molecular markers/genes associated with these major QTLs, after validation, will be useful to undertake marker-assisted breeding for developing better varieties with salinity tolerance.

## 1. Introduction

Chickpea (*Cicer arietinum* L.) is one of the most important dietary grain legumes, and has a small genome size of ~740 Mb [1]. This crop is highly valued for its intrinsic potential for symbiotic nitrogen fixation. It is the primary source of human dietary proteins, vitamins, and essential minerals, and is valuable for food security in the developing world [2]. Due to its high nutrient content, chickpea is ideal for feeding the global population to combat issues related to nutritional food security. The crop is grown on over 17.8 million hectares (ha), producing 17.2 million metric tons (t). India is the world’s leading producer with 11.4 million metric tons, or about 66% of total world’s production [3]. Average chickpea productivity is less than 1 t/ha, far below its global yield potential of 3–4 t/ha in optimum growing conditions. Chickpea productivity is severely affected by several biotic (including Fusarium wilt, Ascochyta blight, Botrytis grey mould, dry root rot, and pod borer) and abiotic (including salinity, drought, and heat) stresses, causing production losses of up to 70% [4,5,6].

Among different abiotic stresses, salinity stress is the second major abiotic stress after drought that limits chickpea productivity and reduces total global production by approximately 8–10% [7,8]. Notably, almost 80 million ha of the world’s arable land is prone to salinity stress [7]. Globally, 20% (45 million ha) of irrigated land and 2% (32 million ha) of dryland are constrained by salinity [9]. In the past few decades, salinity has become one of the major threats to crop productivity, as it influences plant growth at different developmental stages. Salt stress impacts plants by affecting germination, growth, reproduction, and the ability to biologically fix nitrogen [10]. Salinity affects vital physiological functions, hormonal regulation and nutritional balance [11,12], reduce carbon fixation [13], causes flower abortion, reduces flower numbers and pod setting, and eventually limits crop yield [14].

Chickpea is intrinsically salt-sensitive, unlike cereals [7], and salinity causes osmotic imbalance to tissues through specific ion toxicity, nutritional imbalance, and disturbed hormonal interactions, which affects overall growth and development [15,16,17,18]. Increased salt concentration alters grain composition and yield [19,20]. Salinity also increases leaf necrosis and chlorosis, which leads to leaf senescence and reduced photosynthesis in grain legumes [14,21]. While chickpea is sensitive to salt stress, there is a range of variation available for salinity tolerance in germplasm collection [22,23,24].

The development of genetically tolerant cultivars is a preferred strategy for managing salinity stress as plants preserve the quality of the final product. In this direction, mapping of genomic regions/quantitative trait loci (QTLs) responsible for salinity tolerance is a prerequisite for using molecular breeding for developing new cultivars. Several attempts have been made to understand the molecular basis of salt tolerance in many plant species. For instance, QTLs for traits associated with salinity tolerance have been mapped in legumes such as soybean [25], *Medicago truncatula* [26], and cereals such as barley [27] and bread wheat [28]. Advances in next-generation sequencing (NGS) technology are paving the way towards development of high-density SNP-based platforms for genotyping [29]. The development of high-throughput genotyping platforms such as the Axiom*^®^CicerSNP* array has facilitated genetics research in chickpea [30]. Although several mapping studies have identified QTLs for biotic tolerance [31] and drought tolerance [32,33] in chickpea, few studies have identified QTLs for salinity tolerance [34,35]. Furthermore, a very few QTLs have been identified for yield components governing salinity tolerance [36].

Stress tolerance indices, which indicate the comparative performance of genotypes under normal and stress conditions, are the key salinity stress tolerance component traits. Although these traits have been used to map salinity and heat stress tolerance in rice [37,38,39,40], no such report is available in chickpea. Evaluating the crop performance under both control and stress scenarios will serve as a better strategy to identify QTLs and develop crop varieties for enhanced stress tolerance [41]. Such relative performance has practical relevance since genotypes with low yield potential under control conditions often show higher tolerance to stress than high-yielding genotypes. Therefore, it is important to identify QTLs governing salinity stress tolerance for yield and yield components that can be used effectively in marker-assisted breeding in chickpea.

With an objective to dissect the genetic basis of salt tolerance in chickpea, the present study has been undertaken with following objectives: (a) develop a dense genetic map for ICCV 10 (salt stress-tolerant) × DCP 92-3 (salt stress-sensitive) population, (b) identify QTLs for salinity stress tolerance in chickpea, and (c) identify genes underlying major QTLs associated with salinity tolerance component traits in chickpea.

## 2. Results

### 2.1. Phenotypic Variation for Salinity Tolerance Component Traits

RILs, along with parents, were analyzed for their phenotypic performance in control and salt-stressed environments. Salt stress significantly reduced yield in the salinity-sensitive parent (DCP 92-3), being seven-fold more than the salinity-tolerant parent (ICCV 10) across seasons (Table 1). During the 2015–16 and 2016–17 crop seasons, a normal frequency distribution was observed for all traits analyzed, except for stress tolerance index (STI) and stress susceptibility index (SSI) for yield per plant (Supplementary Figures S1 and S2).

The salinity tolerance component traits were subjected to Pearson’s correlation analysis to identify relationships between them. For simplicity, we classified significant correlation values into three different degrees, viz. high, moderate, and low. For instance, a high degree of correlation represented correlation coefficient values between ±0.50 and ±1.0 and was considered to be a strong correlation. A moderate degree of correlation represented coefficient values between ±0.30 and ±0.49, while coefficient values below ±0.29 were classified as low degree of correlation. In this context, we evaluated the correlations between traits that were measured during the 2015–16 season (Table 2, Supplementary Figure S3a). A strong positive correlation was observed between SSI for yield per plant and yield per plant evaluated under control conditions, and between STI for yield per plant and yield per plant under stress conditions. In contrast, SSI for yield per plant and yield per plant measured under salinity stress showed a strong negative correlation. Furthermore, STI for yield per plant displayed a medium positive correlation with pods per plant, 100-seed weight and plant height, all evaluated under salinity stress conditions. STI for 100-seed weight showed a small positive correlation with STI for yield per plant and yield per plant assessed under stress conditions.

We also assessed the correlation between traits evaluated during the 2016–17 crop season (Table 2, Supplementary Figure S3b). Here, STI for yield per plant displayed a strong positive correlation with yield per plant measured under salinity stress. Although SSI for yield per plant was strongly and positively related to yield per plant measured under control conditions, it showed a strong negative correlation with yield per plant measured under stress conditions.

Furthermore, STI for yield per plant showed a positive and moderate correlation with pods per plant and yield per plant, both measured under control scenarios. The SSI for yield per plant was positively and moderately correlated with pods per plant and plant height under control conditions. Furthermore, a small and positive correlation was observed between STI for yield per plant and plant height measured under control, while SSI for yield per plant showed a small and negative correlation with 100-seed weight measured under stress conditions.

### 2.2. High-Density Linkage Map

Of the 50,591 SNP probes on the Axiom^®^*CicerSNP* array, 5123 SNPs were polymorphic between both parents and displayed segregation within the population. In the constructed linkage map, 1856 markers were assigned to eight linkage groups. The integrated map had a total length of 1106.3 cM with an average distance of 0.59 cM between adjacent markers. The number of loci onto eight linkage groups ranged from 56 (CaLG04) to 487 (CaLG07) and the length ranged from 45.6 cM (CaLG08) to 270.7 cM (CaLG06) with a mean value of 138.2 cM (Table 3). The marker density of the linkage groups varied significantly, with the highest marker density on CaLG01 with 327 markers distributed over a distance of 147.7 cM, while CaLG04 had the lowest density with 56 markers distributed over a distance of 87 cM (Table 3).

### 2.3. QTLs for Salinity Tolerance Component Traits

The phenotypic and genotypic data were analyzed for identification of QTLs to understand the genetic basis of salinity tolerance in the RIL mapping population derived from ICCV 10 × DCP 92-3. The QTL analysis was performed for seven yield and yield-related traits, namely, pod number per plant (PPP), 100 seed weight (100SW), yield per plant (YPP), SSI for yield per plant (SSI_YP), SSI for 100 seed weight (SSI_100SW), STI for yield per plant (STI_YP), and STI for 100 seed weight (STI_100SW), and two agronomic traits, i.e., plant height (PH) and branch number per plant (NB). The QTLs that contributed >10% of the phenotypic variation explained (PVE) were considered as major QTLs. Furthermore, if QTL for a given trait in a particular treatment appeared in both years, it was considered as a consistent QTL [32].

#### 2.3.1. QTLs for Yield and Yield-Related Traits

A total of nine major and 12 minor QTLs were detected for seven yield and yield-related traits in the two seasons (2015–16 and 2016–17), as follow:

***Stress susceptible index (SSI) and stress tolerance index (STI):*** SSI and STI are important measures for estimating the effect of salinity tolerance on yield; therefore, QTLs for these traits can be potential targets to improve the salinity response in chickpea. In the case of SSI, four major and two minor QTLs were identified. Among the major QTLs, one major consistent QTL for SSI_YP was identified on CaLG06 in 2015–16 (PVE 12.2%) and 2016–17 (PVE 28.3%), with flanking markers AX-123640392 and AX-123640389. Two other major QTLs for SSI_YP (*qSSIYP3.1*; PVE 10.0%) and SSI_100SW (*qSSI100SW3.1*; PVE 10.1%) on CaLG03 were identified (Table 4). Two minor QTLs for SSI_YP (*qSSIYP6.2*; PVE 8.3%) and SSI_100SW (*qSSI100SW2.1*; PVE 8.9%) on CaLG06 and CaLG02, respectively, were also identified (Table 4).

Similarly, in the case of STI, one major QTL for STI_100SW (*qSTI100SW3.1*; PVE 17.1%) on CaLG03 with flanking markers AX-123659975 and AX-123622699 and two minor QTLs for STI_YP (*qSTIYP5*.1; PVE 8.6% and *qSTIYP6*.1; PVE 8.1%) were identified in the 2016–17 season (Table 4).

***100 seed weight (100SW):*** A total of five QTLs for 100SW (three for saline and two for control treatment) were identified across seasons. One major QTL (*q100SWS3.1*; PVE 13.9%) on CaLG03 with flanking markers AX-123659975 and AX-123622699 in 2016–17, and two minor QTLs (*q100SWS3.1*; PVE 8.8% and *q100SWS2.1*; PVE 7.7%) in 2015–16 for 100SW under saline treatment were identified. Similarly, under control treatment, one major QTL (*q100SWC5.1*; PVE 10.1%) and one minor QTL (*q100SWC7.1*; PVE 7.8%) were identified (Table 4).

***Yield per plant (YPP):*** In the case of YPP, six QTLs, including two major and four minor, were identified under saline and control treatment. Under saline treatment, we could get only one minor QTL (*qYPPS6.1*; PVE 7.1%) on CaLG06 in 2016–17 season (Table 4). However, in 2015–16 under control treatment, two major QTLs namely, *qYPPC6.1* (PVE 13.8%) and *qYPPC5.1* (PVE 10.2%) on CaLG06 and CaLG05, respectively, were identified, of which *qYPPC5.1* was found to be consistent as it appears in 2015–16 as well as in 2016–17 (Table 4). Similarly, three minor QTLs (*qYPPC5.1*; PVE 8.7%, *qYPPC7.1*; PVE 6.5% and *qYPPC4.1*; PVE 6.5%) were detected on CaLG05, CaLG02, and CaLG04, respectively (Table 4).

***Pod number per plant (PPP):*** In the case of PPP, although no major QTL was identified, one minor QTL (*qPPP8.1*; PVE 8.1%) was identified on CaLG08 in the salinity treatment in 2015–16, and one minor QTL with 9.1% PVE was identified on CaLG07 in the control (Table 4).

#### 2.3.2. QTLs for Agronomic Traits

A total of seven QTLs including three major and four minor QTLs were detected for two agronomic traits in two seasons (2015–16 and 2016–17) as follows:

***Plant height (PH):*** In the case of PH, two major QTLs in the 2016–17 season, one each in saline and control treatment, were identified. Under saline treatment, one major QTL (*qPHC5.2*; PVE 10.0%) on CaLG05 flanked by AX-123653281 and AX-123631517 SNP markers was identified. Similarly, under control treatment, one major QTL (*qPHC5.1*; PVE 11.8%) in 2016–17 on CaLG05 flanked by AX-123662454 and AX-123631761 markers and two minor QTLs (*qPHC7.1*; PVE 9.2% and *qPHC6.1*; PVE 8.1%) in 2015–16 were identified (Table 4).

***Number of branches per plant (NB):*** One major QTL (*qNBC8.1*; PVE 12.7) was identified for NB on CaLG08 flanked by AX-123638389 and AX-123638445 markers in 2015–16 under control treatment. This QTL was considered as a consistent QTL as it also appeared in 2016–17 season (Table 4). Two minor QTLs (*qNBC8.2*; PVE 8.9% and *qNBC8.3*; PVE 6.1%) were also identified for NB under control condition on CaLG08 in 2015–16 and 2016–17, respectively. No QTL was identified for NB under the salinity treatment across the seasons (Table 4).

### 2.4. Candidate Genes for Salinity Tolerances

**Figure 1:**
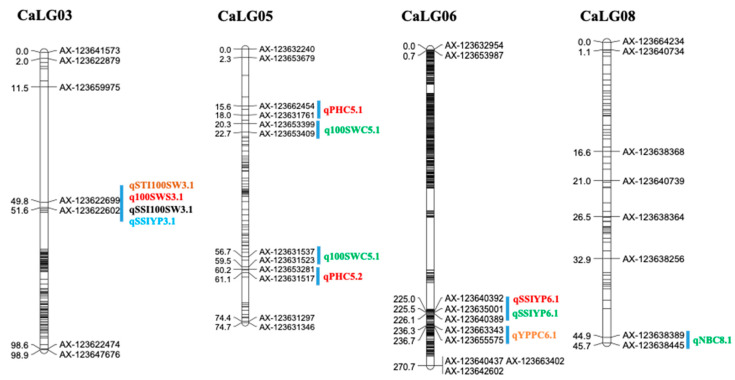
Major quantitative trait loci (QTLs) for various salinity tolerance component traits identified in the DCP 92-3 × ICCV 10 RIL population.

The genes located in the genomic regions for the identified QTLs were extracted. A total of 1121 genes were present in all QTL regions, based on the reference chickpea genome [1]. Of the 1121 genes, 136 putative candidate genes (88 on CaLG03, 25 on CaLG06, 14 on CaLG02, six on CaLG07, and one each on CaLG04 and CaLG08) belong to several gene families, including kinases, genes encoding transcription factors, and ion channels encoding proteins in response to salinity stress, among others. These genomic regions on CaLG03 and CaLG06 are of great interest as they hold QTLs for traits related to yield under salinity (Supplementary Table S1, Figure 1, and Supplementary Figure S4). The major QTL intervals AX-123622602 and AX-123622699 on CaLG03 and AX-123640392 and AX-123655575 on CaLG06 harbored 113 key genes that are reportedly involved in salinity and other abiotic stress tolerances in chickpea and other crops (Supplementary Table S2).

A detailed analysis of QTLs from CaLG03 showed that QTLs for four traits (STI_100SW, SSI_100SW, 100SW, SSI_YP) were flanked by AX-123622602 and AX-123622699 markers. Here, the ~3.3 Mb region between these two markers was selected to identify candidate genes. The ~3.3 Mb region contained 763 predicted genes. Functional annotation of the candidate genes revealed their roles in various biotic and abiotic stress responses. For instance, a MYB transcription factor, which is mainly involved in the regulation of plant growth and development under abiotic stress, such as salinity tolerance, was identified in this region [42,43,44]. Similarly, a C3HC4-type RING zinc finger protein, which reportedly improve and enhances salt tolerance in various crops, was identified [45,46]. An abscisic acid insensitive (ABI) 5, a typical subfamily of proteins belonging to the basic domain/leucine zipper (bZIP) transcription factors, was recently reported to contribute to salt stress tolerance in *Arabidopsis thaliana* via abscisic acid signaling [47]. Notably, the ABI5 gene was also found in the predicted QTL region. The stress-responsive NAC transcription factor family of proteins has been studied extensively for its role in salinity stress tolerance in transgenic plants [48,49]. We identified the presence of the NAC-domain-containing protein in the QTL region. In addition to the above genes, some trait-specific candidate genes, such as E3-ubiquitin-protein ligase, WRKY transcription factor, and zinc finger proteins, were also identified. The gene encoding E3 ubiquitin ligase activity has been shown to regulate grain width and weight in rice [50]. Furthermore, while the differential expression of the WRKY transcription factor gene alters seed size in soybean [51], a zinc finger CCHC-type protein is involved in regulating seed size in *Medicago truncatula* [52]. However, further fine mapping and validation of these genes would be needed to pinpoint the candidate genes regulating traits of interest.

Analysis of the QTLs from the CaLG06 genomic region revealed that QTLs for three traits, namely, STI_YP, SSI_YP, and YPP, were flanked by AX-123640392 and AX-123655575 markers spanning a ~0.1 Mb region. The predicted ~0.1 Mb region encompassed 155 genes. Analysis of the genes underlying the QTL interval identified several potential candidates with a predicted role in salinity stress tolerance. For example, overexpression of cation/proton antiporter genes encoding cellular Na^+^/H^+^ exchanger proteins can upregulate plant performance under salinity stress [53]. A cation/H^+^ antiporter 4-like gene was identified in this region. Further, a plant calmodulin-binding family protein, predicted to be involved in salinity stress responses in plants during early germination, was also detected [54]. Membrane proteins containing a transmembrane domain have been demonstrated to play important roles in conferring salinity tolerance in *Chenopodium quinoa* [55]. In this region, the presence of transmembrane domain proteins is anticipated to be involved in conferring salinity tolerance in the genotypes. Notably, histidine kinases act as sensory molecules and are involved in the transduction of environmental signals in plants, fungi, and prokaryotes. A previous study identified the involvement of histidine kinases in perception or transduction of salt stress signals in *Synechocystis* sp. [56]. The presence of a histidine kinase gene underlying the QTL region is thought to be involved in the perception of salt stress signals and the regulation of salt-inducible genes. We anticipate that further fine mapping and cloning of the candidate genes underlying the major QTL regions will unravel the salinity tolerance mechanism in chickpea.

## 3. Discussion

Recent technological advances have led to the evolution of breeding methodologies that have the potential to accelerate the breeding process [57]. Plant response to abiotic stresses, such as salinity, is a complex trait that is governed by many genes; therefore, breeding approaches for such complex stress tolerance and crop stability have been challenging. The use of marker-assisted selection/marker-assisted breeding in this regard has helped to simplify things to a certain extent. Marker-assisted breeding has successfully produced crops with tolerance to biotic stresses and has increased yield in many crops [57,58,59].

Salinity stress appears to be a polygenic and quantitative trait that is regulated by several genes under diverse environments [60]. To understand the genetic basis of salinity stress, significant efforts have been made to identify QTLs associated with salinity tolerance, with several QTLs mapped on different chromosomes of chickpea by multiple research groups in the last decade. In a study by Vadez et al. [36], QTLs for seed weight, pod number, and harvest index in the salinity treatment were obtained on CaLG07 using a RIL population derived from a cross between salt-tolerant JG62 and salt-sensitive ICCV 2. Similarly, a minor QTL for yield that explained 8% PVE on CaLG07 was identified in chickpea [34]. In another study, two key genomic regions on CaL05 and CaL07, harboring QTLs for yield and other salinity-associated traits, were reported in a RIL population derived from the cross ICCV 2 × JG 11, using SSR and SNP markers [35].

Recent advances in genome-based approaches have endorsed the development of high-throughput approaches for genotyping, enabling the identification of and access to desirable alleles, with different QTLs having the potential to affect desired responses. In the current study, using a reasonably large RIL population, high-density genetic map, and phenotyping under controlled conditions, we identified 28 QTLs for nine traits across seasons and treatments in chickpea. Importantly, two genomic regions on CaLG03 and CaLG06, harboring 10 QTLs for salinity stress indices and yield-related traits, were detected.

Identification of QTLs for relative performance of genotypes under stress and controlled conditions has significant over identification of QTLs based on phenotypic performance in the stress environment alone [41]. QTLs regulating salinity stress tolerance by utilizing stress tolerance indices, which differentiate the performance of crops both under control and stress conditions, have been reported in several crops, but not in chickpea. In rice, genomic regions governing heat stress tolerance [40] and salinity stress tolerance have been successfully mapped using stress indices [37,38,39,61]. In the present study, major QTLs for salinity stress indices SSI_YP, SSI_100SW, STI_100SW, and yield component (100SW) under salinity have been identified. These QTLs, after validation, may play a key role in marker-assisted breeding programs to produce saline-tolerant lines in chickpea. Salt-tolerant genotypes will have lower SSI values, indicating the smaller difference in yield between control and stress treatment, with the reverse being true for susceptible genotypes [37,62].

Identifying the genes involved in the salinity stress response is the first step towards gaining the necessary knowledge for genetics, physiology, and breeding. The application of genomics-based knowledge with breeding platforms is expected to provide useful insights into the molecular responses of plants to salinity. Plants alleviate the salinity stress effects of increased Na^+^ in cells by excluding and sequestering Na^+^. Increased cytosolic Na^+^ concentration is detected and, in turn, the response pathways to stress ion channels, calcium-dependent kinases, histidine kinases, and membrane receptors, etc., activate to regulate gene expression, resulting in the synthesis of compatible solutes to abate the destructive effects of increased Na^+^ ions in the cytoplasm [63]. Plant hormones also play a vital role in regulating plant responses to salinity. Plants induce ABA synthesis to mediate the plant responses through the ABA-dependent signal transduction pathway. To maintain cellular ionic and osmotic homeostasis, ABA induces osmolyte synthesis and stomatal closure in the guard cells, and other related mechanisms leading to the maintenance of cellular ionic and osmotic homeostasis [64,65,66].

The putative candidate genes found in the QTL regions in this study have been experimentally validated for their role in the salinity stress response in several earlier studies in different plant species. For example, genes identified on CaLG03 and CaLG06 that encode several kinases, calcium-dependent protein kinases (CDPKs), mitogen-activated protein kinases (MAPKs), histidine kinases (HKs), sucrose non-fermenting related kinases (SnRK1), transcription factors such as WRKY, basic leucine zipper (bZIP), MYB/MYC, and cation calcium exchanger, were reported to be playing a vital role in the salinity stress response [67,68,69,70]. Similarly, candidate genes coding for proteins related to cation calcium exchanger, cation antiporter 4 (regulates plasma membrane antiporter activity), and potassium channel AKT1 (involved in regulating K^+^/Na^+^ ratio) led to salinity stress tolerance in *Arabidopsis* [71,72]. The transmembrane protein-encoding genes found in the QTL region on CaLG03 are candidate genes for seed weight in chickpea [73].

Among the 113 putative candidate genes found in the QTL regions on CaLG03 and CaLG06, most were involved in osmoregulation, which help plants to cope with salinity and other biotic stresses (Table S3). The identification of probable candidate genes for salinity tolerance on small genomic regions on CaLG03 and CaLG06 make these regions promising for future use in genomics-assisted breeding (GAB) to improve salt and other abiotic stress tolerance in chickpea.

## 4. Materials and Methods

### 4.1. Development of RIL Population

Two diverse chickpea parental lines DCP 92-3 and ICCV 10 that differ in salinity tolerance were used to develop a chickpea mapping population comprising of 201 RILs (F_8_) at the ICAR—Indian Institute of Pulses Research, Kanpur. ICCV 10 is a *desi*-type genotype and is highly tolerant to salinity stress, while DCP 92-3 is susceptible to salinity. The population was advanced using the single seed descent method.

### 4.2. Phenotyping for Salinity Stress

The RIL population was screened for two consecutive years from November to April (2015–16 and 2016–17) at Karnal, Haryana, India located between 29°43′ N longitude and 76°58′ E latitude. Karnal has an average elevation of 240 m from mean average sea level (m.a.s.l) and received total rainfall of 85.8 mm during January, 7.8 mm in March, and 3.4 mm in April, during the crop season 2015–16. The average temperature during cropping season ranged between 11.92 °C and 26.85 °C with minimum temperature of 6.8 °C in December 2015. The initial humidity during seedling stage was 91.87% (max) and 46.06% (min). During 2016–17 season, the average temperature was between 11.82 °C and 27.36 °C, with a minimum temperature of 6.78 °C in the month of January 2017. The total rainfall received during January was 85.8 mm, 7.8 mm in March, and 3.4 mm in April, 2017. The average humidity of 86% (max) and 11% (min) was observed. Overall, the two cropping seasons had cool environmental conditions suitable for crop growth and expression. The experiment was done under control conditions; hence, it did not affect the level of salinity stress in micro-plot. The mapping population was screened using the micro-plot screening method described below.

A total of 201 RILs, along with the parents and Karnal Chana-1 as a positive check, were sown in micro-plots (2 × 2 m^2^) top covered with 2 mm polycarbonate sheet to protect it from unseasonal rains. The experiment was organized in an augmented design to evaluate seed yield and other yield contributing traits in control and saline (ECiw 6 dS/m) conditions at ICAR-Central Soil Salinity Research Institute (CSSRI), Karnal. Initial soil salinity ranged from ECe 0.92–0.97 dS/m. Saline irrigation of ECiw 6 dS/m was applied at 30, 60, and 90 day intervals after sowing. Soil samples were taken from time to time to measure the salinity build-up of ECiw 6 dS/m in the soil.

The genotype response to salt stress was expressed as the stress susceptibility index (SSI; [61]) and stress tolerance index (STI; [37]) using the formulae:

SSI = (1 − Ys/Yp)/SI;SI = 1 − Ȳs/Ȳp [61] and stress tolerance index
STI = Yp × Ys/Ȳp^2^

where Ys and Yp are the yield of genotypes evaluated under saline (stress) and non-saline (nonstress) condition, and Ȳs and Ȳp are the mean yield of all genotypes evaluated under stress and non-stress conditions.

The SSI is a measure of reduction in yield caused by a stress, relative to a favorable environment. Salt-tolerant genotypes would have a lower SSI value, indicating a smaller yield difference with the control than the susceptible genotypes with a larger yield difference. The STI identifies genotypes that produce higher yields in the control and under stress conditions (higher STI value), which is more desirable than only under stress conditions.

### 4.3. High Density Genotyping

Genomic DNA was collected from pooled young leaves of RILs and both parents using the CTAB method [74]. DNA quality was tested in 1% agarose gel and quantified using NanoDrop 8000 spectrophotometer (Thermo Fisher Scientific Inc. Waltham, MA, USA). DNA concentration was adjusted to a minimum of 50 ng/μL. RILs were genotyped using an Axiom^®^*CicerSNP* array with 50,590 probes distributed on all eight linkage groups as described in Roorkiwal et al. [30].

### 4.4. Genetic Map Construction and QTL Analysis

A total of 5123 polymorphic SNP markers were used to construct a genetic linkage map using Join Map v. 4.1 (https://www.kyazma.nl/index.php/JoinMap) [75] as described in [31]. To find QTLs responsible for salinity tolerance, we used multi-seasonal field phenotyping data for yield and agronomical traits associated with salinity tolerance of parental accessions and 201 RILs from the mapping population derived from DCP 92-3 × ICCV 10. SNP genotyping data, obtained from Axiom^®^*CicerSNP* array-based SNP genotyping, was correlated with the aforementioned phenotyping data using Windows QTL Cartographer ver. 2.5 [76] (composite interval mapping at significant LOD (logarithm of odds) threshold >3.0 at a *p* < 0.05) as per earlier studies [77,78].

### 4.5. Mining of Candidate Genes

To identify candidate genes, present within the QTL region, flanking markers were subjected to BLAST against chickpea reference genome assembly, and candidate genes in the QTL regions were retrieved.

### 4.6. Statistical Analysis

Pearson’s correlation analysis was conducted using the “Hmisc” package in R. Frequency distributions of the component traits within the RIL population were analyzed and plotted with “UsingR” package within the R environment.

## 5. Conclusions

The present study, using a DCP 92-3 × ICCV 10 RIL mapping population and 50K SNP genotyping array, identified two genomic regions that harbor QTLs for salinity tolerance in a ~3.3 Mb region on CaLG03 and ~0.1 Mb region on CaLG06 with major QTLs for yield and salinity tolerance. The genes present in the identified QTL regions in this study reportedly play a key role in salinity tolerance. Further sequencing and functional validation are required to identify the candidate genes in these QTL regions responsible for salt tolerance. Nonetheless, the high-density linkage map, major QTLs, and genomic regions identified in this study can be used in GAB, after suitable markers are developed and validated, to breed high-yielding salinity-tolerant chickpea varieties.

## Figures and Tables

**Table 1:** Phenotypic variation for the parameters evaluated in the control and salinity treatments in the ICCV 10 × DCP 92-3 RIL population.

S. No.			RILs						ICCV 10	DCP 92-3
	Traits	Treatment	Minimum	Maximum	Range	Mean	SD *	CV **		
**Karnal 2015–16**										
1	PH (cm)	Control	29	41	12	37.1	6.2	0.2	46.3	51.0
2		Saline	27.5	29.5	2	27.3	4.5	0.2	31.0	22.0
3	NB	Control	8	9	1	9.2	2.7	0.3	8.6	6.5
4		Saline	4	5.5	1.5	4.1	1.3	0.3	5.0	3.0
5	PPP	Control	61	87	26	65.1	21.9	0.3	84.0	32.0
6		Saline	12	35	23	22.7	12.3	0.5	39.0	10.0
7	100SW (g)	Control	12	12.8	0.8	13.7	1.7	0.1	11.4	12.9
8		Saline	6.3	10	3.7	8.7	2.2	0.3	12.2	10.2
9	YPP (g)	Control	2.0	27.2	25.2	11.1	4.8	0.4	17.2	18.8
10		Saline	0.29	11.8	11.5	2.8	2.2	0.8	7.2	1.0
11	SSI_YP	NA	1	1	0	1.0	0.1	0.1	1.0	1.0
12	STI_YP	NA	0	0.1	0.1	0.1	0.1	1.1	0.2	0.0
13	SSI_100SW	NA	0.3	1	0.7	0.7	0.3	0.4	−0.1	0.4
14	STI_100SW	NA	0.4	0.6	0.2	0.6	0.2	0.3	0.7	0.7
**Karnal 2016–17**										
1	PH (cm)	Control	48.3	54.7	6.4	46.5	8.7	0.2	58.67	53.33
2		Saline	27.5	39.3	11.8	31.6	10.7	0.3	29.00	22.00
3	NB	Control	8.1	9	0.9	9.8	3.3	0.3	7.33	7.00
4		Saline	2.3	4.3	2	2.6	7.3	2.9	2.33	2.00
5	PPP	Control	14	164	150	50.9	21.8	0.4	56.00	45.00
6		Saline	19.7	30	10.3	20.9	11.1	0.5	18.33	27.33
7	100SW (g)	Control	11.6	13.7	2.1	13.7	1.7	0.1	11.35	12.95
8		Saline	4.4	8.8	4.4	5.9	7.7	1.3	13.10	7.15
9	YPP (g)	Control	2.6	26.6	24	11.6	4.9	0.4	10.20	11.47
10		Saline	0.18	7.8	7.6	1.3	0.9	9.1	1.09	1.19
11	SSI_YP	NA	0.998	1.011	0.013	1.0	0.0	0.0	1.65	1.57
12	STI_YP	NA	0.013	0.028	0.015	0.0	0.0	0.7	0.20	0.18
13	SSI_100SW	NA	0.7	1.2	0.5	1.0	0.3	0.3	0.21	0.61
14	STI_100SW	NA	0.3	0.7	0.4	0.5	0.3	0.2	1.05	1.05

* Standard deviation. ** Coefficient of variation (PH: plant height; NB: branch number per plant; PPP: pods number per plant; 100SW: 100 seed weight; YPP: yield per plant; SSI_YP: SSI for yield per plant; STI_YP: STI for yield per plant; SSI_100SW: SSI for 100 seed weight; STI_100SW: STI for 100 seed weight; NA: not applicable).

**Table 2:** Pearson correlation analysis for salinity tolerance component traits evaluated during the 2015–16 and 2016–17 seasons.

	PH_C	NB_C	PPP_C	100SW_C	YPP_C	PH_S	NB_S	PPP_S	100SW_S	YPP_S	SSI_YP	STI_YP	SSI_100SW	STI_100SW
	**2015–16**													
**PH_C**	1													
**NB_C**	0.11 ns	1												
**PPP_C**	0.07 ns	−0.05 ns	1											
**100SW_C**	−0.12 ns	−0.04 ns	−0.10 ns	1										
**YPP_C**	0.27 ***	−0.23 **	−0.03 ns	−0.03 ns	1									
**PH_S**	−0.01 ns	0.00 ns	−0.05 ns	−0.10 ns	−0.07 ns	1								
**NB_S**	0.01 ns	−0.07 ns	0.03 ns	−0.09 ns	−0.07 ns	0.24 **	1							
**PPP_S**	0.00 ns	−0.17 *	0.13 ns	−0.07 ns	0.08 ns	0.31 ***	0.37 ***	1						
**100SW_S**	0.18 *	−0.20 *	−0.04 ns	0.22 **	0.06 ns	−0.02 ns	0.03 ns	0.17 *	1					
**YPP_S**	0.01 ns	−0.09 ns	0.04 ns	−0.02 ns	−0.14 ns	0.44 ***	0.15 ns	0.43 ***	0.33 ***	1				
**SSI_YP**	0.20 **	−0.13 ns	0.00 ns	−0.07 ns	0.52 ***	−0.20 *	−0.02 ns	−0.14 ns	−0.19 *	−0.55 ***	1			
**STI_YP**	0.16 *	−0.24 **	0.06 ns	−0.02 ns	0.42 ***	0.32 ***	0.11 ns	0.43 ***	0.33 ***	0.68 ***	0.02 ns	1		
**SSI_100SW**	−0.22 **	0.19 *	0.00 ns	0.25 **	−0.08 ns	−0.03 ns	−0.08 ns	−0.19 *	−0.88 ***	−0.34 ***	0.15 *	−0.32 ***	1	
**STI_100SW**	0.12 ns	−0.17 *	−0.08 ns	0.57 ***	0.07 ns	−0.06 ns	−0.02 ns	0.10 ns	0.91 ***	0.24 **	−0.15 *	0.27 ***	−0.62 ***	1
	**2016–17**													
**PH_C**	1													
**NB_C**	−0.10 ns	1												
**PPP_C**	0.67 ***	−0.19 **	1											
**100SW_C**	−0.10 ns	−0.03 ns	−0.06 ns	1										
**YPP_C**	0.67 ***	−0.19 **	0.67 ***	−0.06 ns	1									
**PH_S**	0.07 ns	0.10 ns	0.01 ns	−0.03 ns	0.09 ns	1								
**NB_S**	0.05 ns	−0.18 *	0.07 ns	0.19 **	0.18 *	0.33 ***	1							
**PPP_S**	0.12 ns	−0.13 ns	0.14 *	0.00 ns	0.16 *	0.47 ***	0.48 ***	1						
**100SW_S**	−0.06 ns	−0.15 *	0.05 ns	0.06 ns	0.01 ns	0.11 ns	0.08 ns	0.07 ns	1					
**YPP_S**	−0.15 *	−0.13 ns	0.01 ns	0.00 ns	−0.06 ns	0.09 ns	0.00 ns	0.10 ns	0.28 ***	1				
**SSI_YP**	0.45 ***	0.00 ns	0.31 ***	−0.13 ns	0.52 ***	−0.06 ns	0.09 ns	0.02 ns	−0.17 *	−0.78 ***	1			
**STI_YP**	0.25 ***	−0.22 **	0.38 ***	−0.02 ns	0.50 ***	0.13 ns	0.11 ns	0.17 *	0.29 ***	0.78 ***	−0.29 ***	1		
**SSI_100SW**	0.01 ns	0.13 ns	−0.07 ns	0.31 ***	−0.05 ns	−0.11 ns	−0.03 ns	−0.07 ns	−0.92 ***	−0.26 ***	0.10 ns	−0.29 ***	1	
**STI_100SW**	−0.09 ns	−0.15 *	0.02 ns	0.45 ***	−0.02 ns	0.09 ns	0.13 ns	0.07 ns	0.91 ***	0.25 ***	−0.22 **	0.25 ***	−0.69 ***	1

*** *p* < 0.001; ** *p* < 0.01; * *p* < 0.05; ns, non-significant. (PH: plant height; NB: branch number per plant; PPP: pods number per plant; 100SW: 100 seed weight; YPP: yield per plant; SSI_YP: SSI for yield per plant; STI_YP: STI for yield per plant; SSI_100SW: SSI for 100 seed weight; STI_100SW: STI for 100 seed weight; S: under saline condition; C: under control condition).

**Table 3:** Distribution of markers on the eight linkage groups (LGs) of the chickpea genetic map for the ICCV 10 × DCP 92-3 RIL population.

S. No.	LGs	Genetic Distance (cM)	Number of Markers Mapped	Inter Marker Distance (cM)
1	CaLG1	147.69	327	0.5
2	CaLG2	120.4	192	0.6
3	CaLG3	98.9	158	0.6
4	CaLG4	87	56	1.6
5	CaLG5	74	86	0.9
6	CaLG6	270.75	476	0.6
7	CaLG7	262	487	0.5
8	CaLG8	45.6	74	0.6
	**Total**	**1106.34**	**1856**	**0.6**

**Table 4:** Summary of the major and minor QTLs for various salinity tolerance component traits.

Trait Name	QTL Name	Year	Treatment	LG	Position (cM)	Marker Interval	LOD Value	PVE (%)	Additive Effect	Allele-Contributing Parent
**Yield and Yield-Related Traits**										
SSI_YP	*qSSIYP6.1*	2016–17	–	CaLG06	226.1	AX-123640392–AX-123640389	5.7	28.4	0.1	DCP92-3
	*qSSIYP6.1*	2015–16	–	CaLG06	226.1	AX-123640392–AX-123640389	4.8	12.2	0.1	DCP92-3
	*qSSIYP3.1*	2016–17	–	CaLG03	50.79	AX-123659975–AX-123622699	5.3	10.0	0	DCP92-3
	*qSSIYP6.2*	2016–17	–	CaLG06	260.98	AX-123635094–AX-123635091	3.8	8.3	0	DCP92-3
SSI_100SW	*qSSI100SW3.1*	2016–17	–	CaLG03	50.79	AX-123659975–AX-123622699	4.6	10.1	0.1	DCP92-3
	*qSSI100SW2.1*	2015–16	–	CaLG02	62.91	AX-123659415–AX-123620733	3.7	8.9	0.1	DCP92-3
STI_YP	*qSTIYP5.1*	2016–17	–	CaLG05	20.31	AX-123653399–AX-123653409	4.8	8.6	0	DCP92-3
	*qSTIYP6.1*	2016–17	–	CaLG06	268.65	AX-123640437–AX-123655585	4.3	8.1	0	DCP92-3
STI_100SW	*qSTI100SW3.1*	2016–17	–	CaLG03	50.79	AX-123659975–AX-123622699	8.7	17.1	−0.1	ICCV10
100SW	*q100SWS3.1*	2016–17	Saline	CaLG03	50.79	AX-123659975–AX-123622699	6.9	13.9	−0.8	ICCV10
	*q100SWS3.1*	2015–16		CaLG03	56.21	AX-123641496–AX-123622502	3.1	8.8	−0.7	ICCV10
	*q100SWS2.1*	2015–16		CaLG02	39.16	AX-123620430–AX-123659350	3.4	7.7	−0.6	ICCV10
	*q100SWC5.1*	2015–16	Control	CaLG05	58.7	AX-123631523–AX-123631537	3.2	10.1	−0.9	ICCV10
	*q100SWC7.1*	2015–16		CaLG07	92.67	AX-123636120–AX-123636108	3	7.8	0.8	DCP92-3
YPP	*qYPPS6.1*	2016–17	Saline	CaLG06	270.65	AX-123635072–AX-123640437	3.5	7.1	0.2	DCP92-3
	*qYPPC6.1*	2015–16	Control	CaLG06	236.66	AX-123655575–AX-123663343	6.5	13.8	−0.8	ICCV10
	*qYPPC5.1*	2015–16		CaLG05	20.31	AX-123653409–AX-123653399	4.7	10.2	7.8	DCP92-3
	*qYPPC5.1*	2016–17		CaLG05	20.31	AX-123653399–AX-123653409	4.9	8.7	7.4	DCP92-3
	*qYPPC7.1*	2015–16		CaLG02	120.16	AX-123620346–AX-123620222	3.2	6.5	6.7	DCP92-3
	*qYPPC4.1*	2016–17		CaLG04	87.63	AX-123630936–AX-123652553	3.7	6.5	6.5	DCP92-3
PPP	*qPPP8.1*	2015–16	Saline	CaLG08	36.06	AX-123638292–AX-123664233	3.3	8.1	3.6	DCP92-3
**Agronomic traits**										
PH	*qPHC5.2*	2016–17	Saline	CaLG05	60.33	AX-123653281–AX-123631517	4.1	10.0	3.6	DCP92-3
	*qPHC5.1*	2016–17	Control	CaLG05	16.55	AX-123662454–AX-123631761	6.1	11.8	3.9	DCP92-3
	*qPHC7.1*	2015–16		CaLG07	230.14	AX-123635844–AX-123655878	3.8	9.2	8.9	DCP92-3
	*qPHC6.1*	2015–16		CaLG06	44.23	AX-123654072–AX-123633446	3.4	8.1	−4.9	ICCV10
NB	*qNBC8.1*	2015–16	Control	CaLG08	44.91	AX-123638389–AX-123638445	5.5	12.7	−1.7	ICCV10
	*qNBC8.2*	2015–16		CaLG08	10.22	AX-123657789–AX-123638459	4	8.9	1.4	DCP92-3
	*qNBC8.3*	2016–17		CaLG08	44.91	AX-123638389–AX-123638445	3.5	6.1	−1.4	ICCV10

(SSI_YP: SSI for yield per plant; SSI_100SW: SSI for 100 seed weight; STI_YP: STI for yield per plant; STI_100SW: STI for 100 seed weight; 100SW: 100 seed weight; YPP: yield per plant; PPP: pods number per plant; PH: plant height; NB: number of branches per plant).

## Supplementary Materials

Supplementary materials can be found at https://www.mdpi.com/1422-0067/21/14/5058/s1. Table S1: Summary of major and minor QTLs identified on CaLG03 and CaLG06. Table S2: List of genes present in identified QTL regions. Table S3: List of putative candidate genes associated with salinity stress response on CaLG03 and CaLG06. Figure S1: Frequency distribution of salinity tolerance component traits evaluated in 2015–16. PH: plant height; NB: number of branches per plant; PPP: pod number per plant; 100SW: 100 seed weight; YPP: yield per plant; SSI-YP: SSI for yield per plant; STI-YP: STI for yield per plant; SSI-100SW: SSI for 100 seed weight; STI-100SW: STI for 100 seed weight. Figure S2: Frequency distribution of salinity tolerance component traits evaluated in 2016–17. PH: plant height; NB: branch number per plant; PPP: pod number per plant; 100SW: 100 seed weight; YPP: yield per plant; SSI-YP: SSI for yield per plant; STI-YP: STI for yield per plant; SSI-100SW: SSI for 100 seed weight; STI-100SW: STI for 100 seed weight. Figure S3: Correlation matrices for salinity tolerance component traits evaluated across two seasons. Correlation matrix for (a) 2015–16 season, and (b) 2016–17 season. PH: plant height; NB: branch number per plant; PPP: pod number per plant; 100SW: 100 seed weight; YPP: yield per plant; SSI-YP: SSI for yield per plant; STI-YP: STI for yield per plant; SSI-100SW: SSI for 100 seed weight; STI-100SW: STI for 100 seed weight. Figure S4: Major QTLs for salinity tolerance component traits identified in DCP 92-3 × ICCV 10 RIL population. (a) QTLs on CaLG06 in 2015–16, (b) QTLs on CaLG03 in 2015–16, (c) QTLs on CaLG06 in 2016–17, and (d) QTLs on CaLG03 in 2016–17.
