# Supplementary figures and images for: Genetic Dissection and Identification of Candidate Genes for Salinity Tolerance Using Axiom®CicerSNP Array in Chickpea
Source: Int J Mol Sci. 2020 Jul 17;21(14):5058. doi: 10.3390/ijms21145058 (PMC7404205; doi:10.3390/ijms21145058)

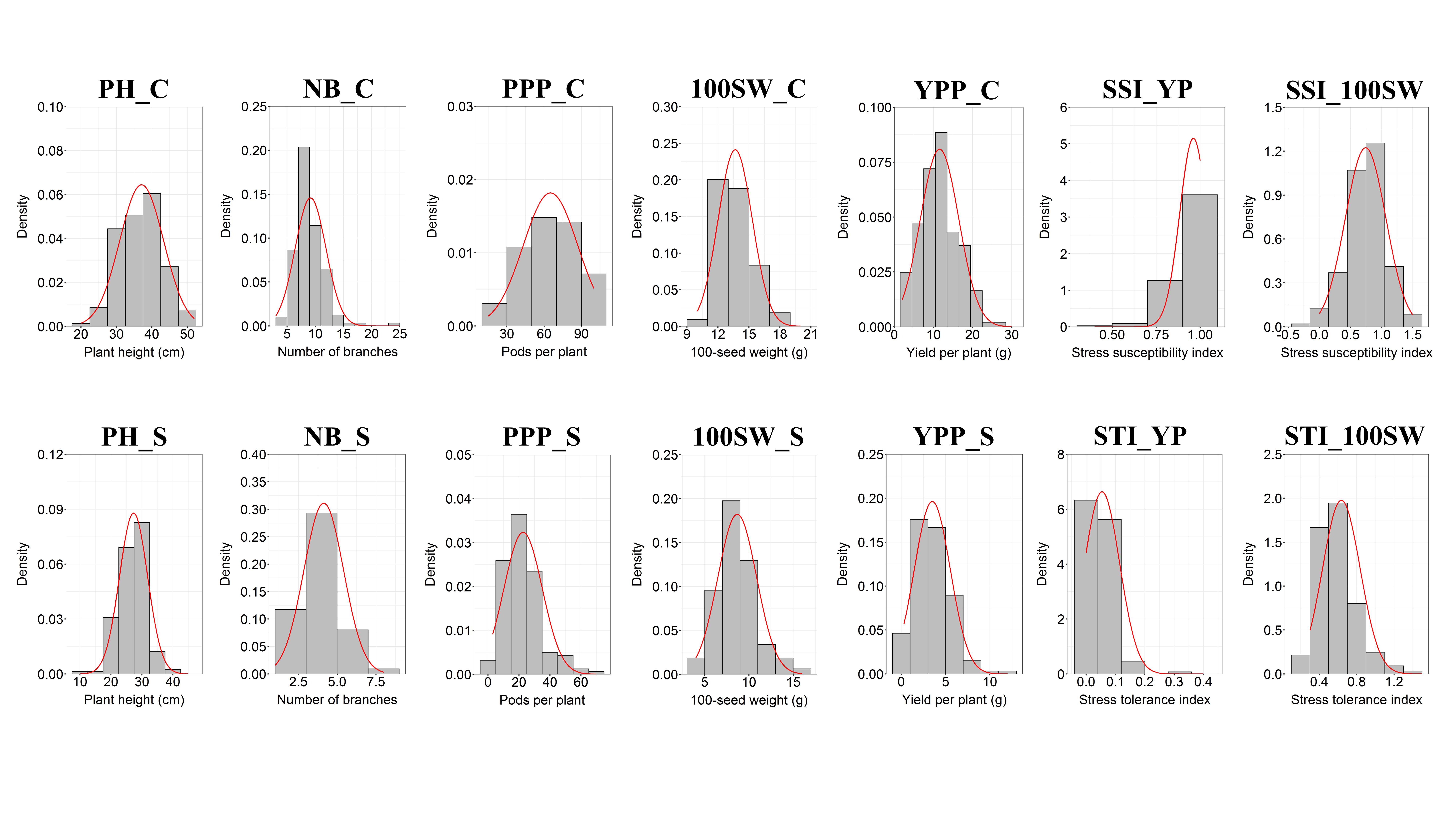

Supplement: Supplementary file 1 [file ijms-21-05058-s001.zip › Supplementary Tables and Figures 17072020/Supplementary Fig. 1.TIF]

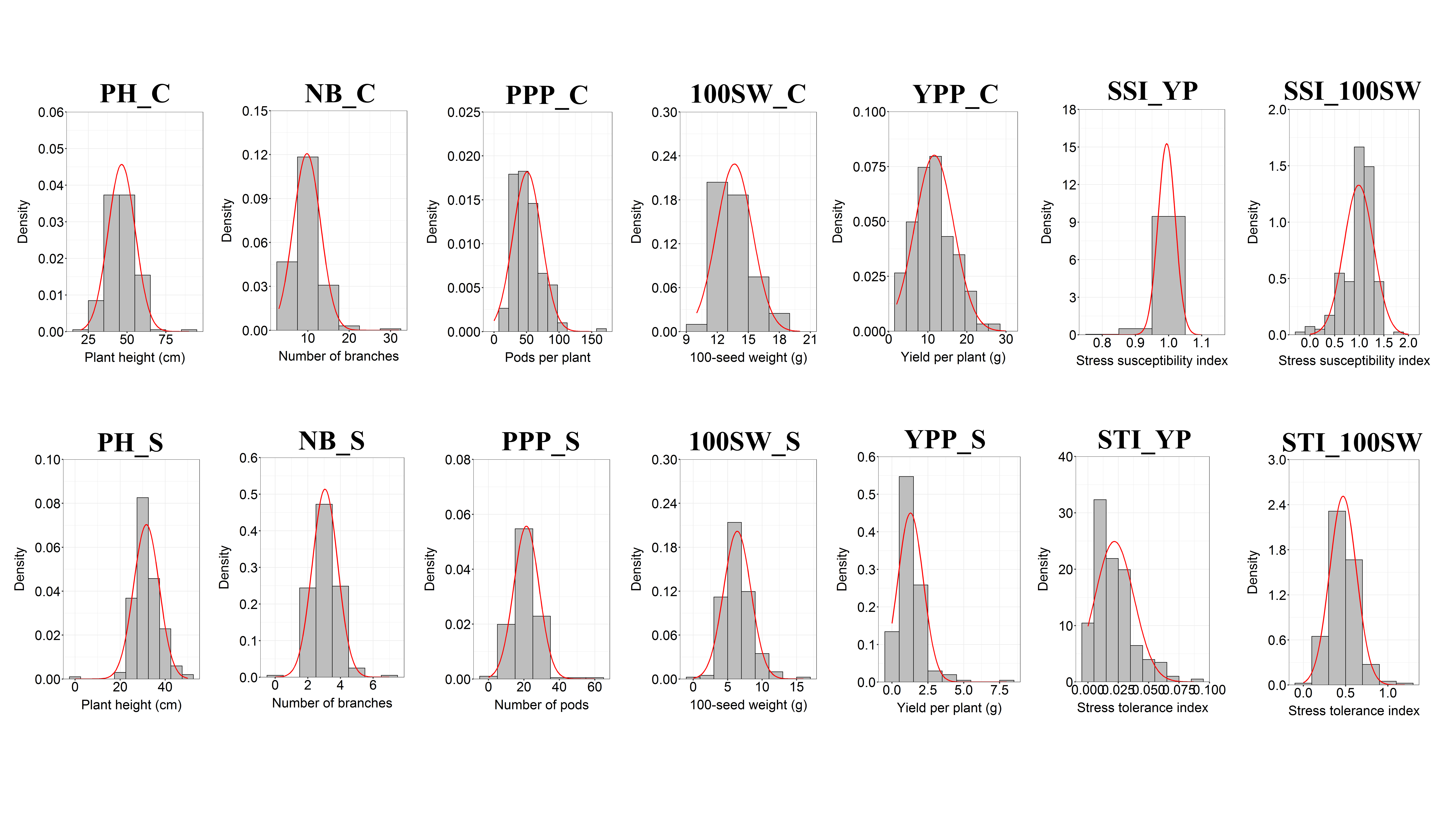

Supplement: Supplementary file 1 [file ijms-21-05058-s001.zip › Supplementary Tables and Figures 17072020/Supplementary Fig. 2.TIF]

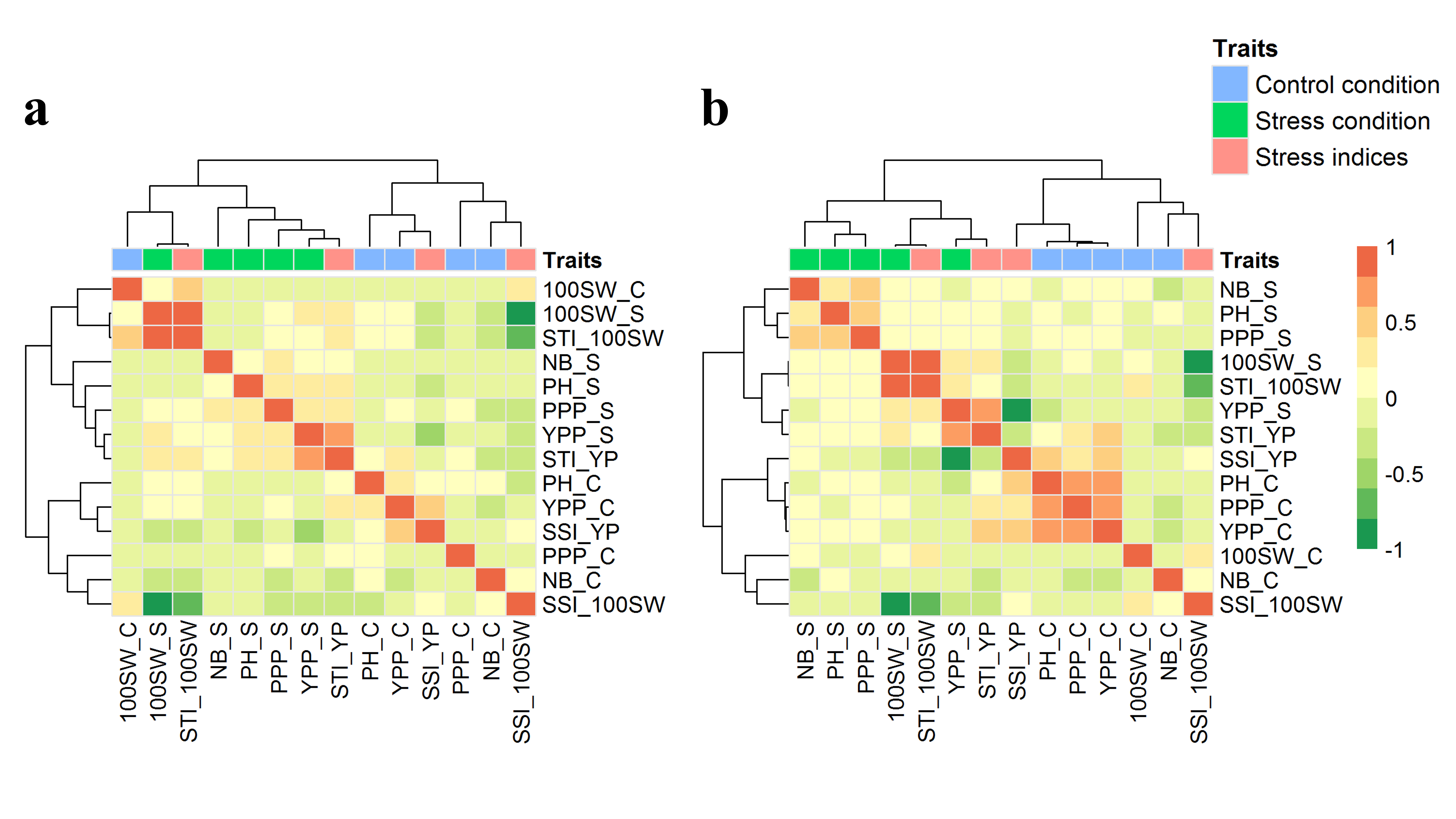

Supplement: Supplementary file 1 [file ijms-21-05058-s001.zip › Supplementary Tables and Figures 17072020/Supplementary Fig. 3.tif]

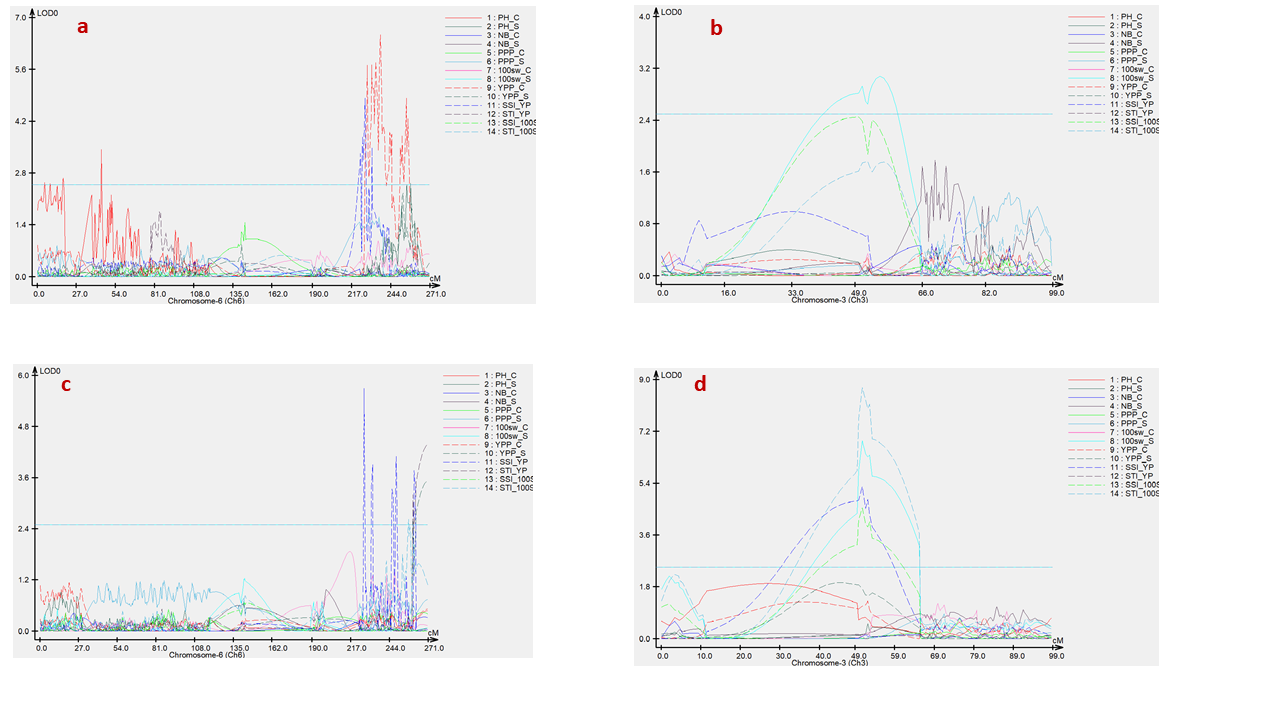

Supplement: Supplementary file 1 [file ijms-21-05058-s001.zip › Supplementary Tables and Figures 17072020/Supplementary Fig. 4.tif]
